# Visceral And Subcutaneous Fat as Predictors of Diabetes Mellitus: A Computed Tomography Scan-Based Study in Southern Indian Rural Population

**DOI:** 10.7759/cureus.86144

**Published:** 2025-06-16

**Authors:** Soumya Chincholikar, Anil K Sakalecha, Goravimakalahalli Srinivasareddy Hemanth Kumar, Shantala Sawkar, Vidya Sagar C R

**Affiliations:** 1 Radiodiagnosis, Sri Devaraj Urs Medical College, Kolar, IND; 2 Medicine, Sri Devaraj Urs Medical College, Kolar, IND

**Keywords:** adiposity, bmi, computed tomography diagnostic, diabetes, fat, hemoglobin, subcutaneous fat accumulation, subcutaneous fat area, visceral fat accumulation, visceral fat area

## Abstract

Introduction

Type 2 diabetes mellitus is constantly increasing globally, and obesity is deemed a “high-risk factor” for it. Visceral fat plays a significant role in the development and progression of metabolic syndrome. However, the effect of subcutaneous fat accumulation (SFA) remains unclear and warrants further investigation. Although imaging modalities such as magnetic resonance imaging, computed tomography (CT), and dual-energy X-ray absorptiometry are commonly employed to evaluate visceral fat, their high cost and limited accessibility restrict their routine clinical use. This highlights the urgent need for simple, cost-effective, reliable, and widely accessible diagnostic tools.

Material & Methods

A retrospective study was conducted on 240 individuals (120 patients with diabetes and 120 without diabetes) aged 18-75 years. Patients who underwent plain CT scans of the abdomen, pelvis, kidney, ureter, and bladder at the L3 vertebral level were considered for this study. The study was performed using a Siemens 128-slice SOMATOM Go-top CT machine. Data were obtained using the SYNAPSE software. Visceral and subcutaneous fat volumes were measured using a fat analyzer by adjusting the measurement plane to the level of the umbilicus.

Results

The mean ages were recorded as 46.47 and 42.60 years in patients with diabetes and without diabetes, respectively, with male predominance (53.33%) among patients with diabetes and female predominance (51.67%) among patients without diabetes. The mean HbA1c and BMI values were 6.57% and 25.63% in patients with diabetes and 5.38 kg/m^2^ and 23.62 kg/m^2^ in patients without diabetes, respectively. The mean visceral fat accumulation (VFA) was recorded to be 3075.72 cm^3^ and 2042.54 cm^3^ in patients with and without diabetes, whereas the mean SFA was recorded to be 3309.39 cm^3^ and 1795.75 cm^3^ in patients with and without diabetes, respectively; and this was found to be statistically significant.

Conclusion

We recorded significant differences in VFA and SFA between individuals with and without diabetes, illustrating the alterations that patients with diabetes undergo. Over the years, extensive research on the assessment of VFA has been conducted and it has proven to be a reliable marker. However, there is a lack of similar evidence for SFA. Therefore, we recommend using it as an adjunct to VFA.

## Introduction

Globally, approximately 13% of adults are obese, which is characterised by improper fat distribution and frequently manifests as abdominal obesity (notably central adiposity). Abdominal obesity is the most common type of obesity and an important indicator of the risk of diabetes mellitus [1-4]. Type 2 diabetes mellitus (T2DM) is a global health issue. According to 2017 data, approximately 451 million adults aged 18-99 years are living with diabetes, and this number is projected to increase to 693 million by 2045. It accounts for nearly 49.7% of the global adult population and encompasses both diagnosed and undiagnosed cases. In 2017 alone, diabetes was estimated to be responsible for approximately five million deaths globally among individuals aged 20-99 years [3,4]. A bidirectional relationship has often been proposed between T2DM and visceral fat accumulation (VFA) because individuals with T2DM commonly exhibit elevated visceral adiposity. However, this association is not consistent across all patients, as some individuals with T2DM do not show a markedly increased visceral fat area. This variability may be influenced by several factors, including ethnicity, genetic predisposition, and differences in patterns of body composition. Notably, South Asians, including the rural Indian population, tend to accumulate visceral fat at lower BMI levels compared to Western populations, and often exhibit higher metabolic risk despite a normal body weight. Additionally, distinct dietary habits and physical activity levels in rural regions may further modulate fat distribution. These factors underscore the importance of studying fat distribution specifically in a rural South Indian cohort. This variability raises uncertainty regarding the link between glycated hemoglobin (HbA1c) levels and visceral fat area, contributing to the ongoing debate and ambiguity in the literature [5]. VFA, a defining feature of abdominal obesity, plays a key role in the development and progression of metabolic syndrome (MetS), which encompasses conditions such as diabetes, dyslipidemia, and hypertension [3,6]. Central obesity, characterized by increased VFA, is associated with oxidative stress and insulin resistance, both of which are critical contributors to the pathogenesis of T2DM.

Recent evidence suggests that poorly-controlled diabetes is often associated with a higher BMI than in well-managed cases. Conversely, when diabetes is compounded by pancreatic cancer, patients may experience a decline in BMI and elevated HbA1c levels. While BMI is a convenient and widely-used tool for screening large populations, it is a poor marker of adiposity and fails to capture the complexities of fat distribution and metabolic risk, particularly visceral fat accumulation, which plays a more critical role in T2DM and cardiovascular disease. Moreover, the concept of sarcopenic obesity, characterized by increased fat mass with concurrent loss of skeletal muscle, adds further complexity, particularly in older adults and certain ethnic groups, including South Asians. This condition can mask true metabolic risk under a seemingly normal BMI, highlighting the limitations of BMI as a solitary screening tool for central adiposity and related disorders [7].

From a clinical perspective, HbA1c values serve as screening indicators of T2DM, and high HbA1c levels may inadvertently lead to weight loss. Subcutaneous fat may offer protective effects against metabolic disorders in contrast to visceral fat, which is closely linked to increased cardiometabolic risk. Subcutaneous fat, particularly in the gluteofemoral region, is believed to exert protective metabolic effects by serving as a metabolic sink that safely stores excess lipids, preventing their ectopic deposition in organs such as the liver, pancreas, and muscle. This contrasts with visceral fat, which is more metabolically active and pro-inflammatory, secreting higher levels of adipokines and cytokines (e.g., IL-6, TNF-α) that contribute to insulin resistance, systemic inflammation, and endothelial dysfunction. These distinct endocrine and immunological profiles may explain why greater subcutaneous fat accumulation has been associated with improved insulin sensitivity and lower cardiometabolic risk, particularly in women and younger individuals. Nonetheless, the protective role of subcutaneous fat remains complex and may vary by age, sex, ethnicity, and overall body composition. Considering the distinct effects of the VFA and subcutaneous fat accumulation (SFA) on cardiovascular health, the overall distribution of body fat appears to be more informative than the individual assessment of VFA or SFA alone. Notably, the VFA-to-SFA ratio (V/S ratio) has been identified as a more robust predictor of cardiometabolic risk than the VFA alone. For instance, a study involving patients with T2DM demonstrated that a higher V/S ratio was significantly associated with an increased risk of cardiovascular disease, whereas VFA, SFA, and BMI alone were not strongly associated with such outcomes [8]. However, most existing data on the V/S ratio originate from Western populations, and its utility in South Asians-who tend to have higher visceral fat even at lower BMI levels-remains poorly validated. This gap underscores the relevance of our study in evaluating fat distribution metrics within a rural South Indian cohort. However, the role of SFA in metabolic health remains controversial. Therefore, measuring visceral fat accumulation is critical for identifying individuals at an increased risk of diabetes and other cardiovascular diseases.

Magnetic resonance imaging, computed tomography (CT), and dual-energy X-ray absorptiometry are the gold standards for the clinical assessment of visceral adiposity; however, they are not suitable for large populations due to high testing costs, prolonged exposure to harmful radiation, and complicated procedures. Therefore, there is a dearth of indices and diagnostics that provide a reliable assessment of visceral obesity, which could be useful in determining the risk of diabetes mellitus [9].

The goal of this study was to examine the relationship between VFA and SFA, as measured by CT scans and HbA1c, in order to improve T2DM management.

## Materials and methods

This retrospective study was designed and conducted on 240 individuals who visited the Department of Radiodiagnosis at Sri Devaraj Urs Medical College, Kolar, Karnataka, India. The participants were aged 18-75 years. Patients who underwent plain CT scans of the abdomen and pelvis, and CT of the kidney, ureter, and bladder at the L3 vertebral level were considered for this study. The CT scans evaluated in this study were performed for non-metabolic clinical indications such as renal colic, suspected urolithiasis, abdominal pain, or trauma. Importantly, these indications were not matched between the diabetic and non-diabetic groups. However, all included participants underwent non-contrast abdominal and pelvic CT imaging that allowed for standardized fat quantification at the L3 level. We acknowledge that using scans done for unrelated clinical purposes may introduce selection bias. To mitigate this, patients with known metabolic or endocrine disorders other than T2DM were excluded, and demographic characteristics such as age and sex were matched between the groups as closely as possible. The study was performed using a SIEMENS 128-slice SOMATOM Go-top CT machine (Siemens Healthineers, Erlangen, Germany). Data were obtained using the SYNAPSE software (FUJIFILM Healthcare Corporation, Tokyo, Japan). Visceral and subcutaneous fat volumes (in cubic centimeters) were quantified using automated segmentation software integrated with SYNAPSE, after positioning the measurement plane at the level of the umbilicus using a fat analyzer. Fat was classified based on standard Hounsfield Unit thresholds (−190 to −30 HU). Measurements were recorded at the umbilicus level, approximating the L3 vertebral level. This study was conducted retrospectively. The participants were not exposed to additional radiation from CT scans, specifically for this study.

A total of 240 patients were enrolled in the study and were equally distributed between two groups: Group A consisted of 120 individuals diagnosed with T2DM, whereas Group B included 120 individuals without diabetes. The non-diabetic status in Group B was confirmed by the absence of a documented diagnosis of T2DM in medical records, no history of antidiabetic medication use, and an HbA1c value below 5.7% recorded within the previous three months. 

Data collection was performed after obtaining ethical approval from the Institutional Ethics Committee of Sri Devaraj Urs Medical College, Kolar, Karnataka, India (approval number: SDUAHER/KLR/R&D/IEC/23/2024-25).

The inclusion criteria were individuals aged between 18 and 75 years who underwent CT scans for various clinical indications. In Group A, a confirmed diagnosis of T2DM was required. Participants were excluded if they had an estimated glomerular filtration rate of less than 15 mL/min/1.73 m², were undergoing hemodialysis, were pregnant, or had a diagnosis of type 1 diabetes mellitus (T1DM). Additional exclusion criteria included the use of medications known to significantly influence fat distribution or glucose metabolism (e.g., long-term corticosteroids and certain antipsychotics) within the past six months, as well as the presence of other metabolic disorders such as Cushing’s syndrome or polycystic ovary syndrome that could confound the relationship between fat distribution and diabetes mellitus. Medication history was verified using institutional electronic medical records and patient charts to ensure accuracy in identifying antidiabetic drug use or medications affecting fat distribution.

Statistical analysis

The sample size was estimated based on the area under the receiver operating characteristic (ROC) curve (AUC), as referenced in a study by Yokokawa et al. [3], which investigated the association between visceral or subcutaneous fat accumulation and diabetes mellitus in Japanese individuals. Using the formula a = ZAUC x 1.414 and substituting an AUC value of 0.61 with a margin of error of 5%, the calculated sample size was 239. Subsequently, this was rounded to the nearest multiple of 10, yielding a final sample size of 240 participants. Data were analyzed using IBM SPSS Statistics for Windows, Version 25 (Released 2017; IBM Corp., Armonk, New York, United States). Non-parametric statistical tests were used due to non-normal distribution of the data. The Mann-Whitney U test was applied to compare differences in VFA and SFA between diabetic and non-diabetic groups within each gender and age category. The Kruskal-Wallis H test was used to assess differences across multiple age groups within the diabetic and non-diabetic populations. Test statistics (U-values and H-values) and corresponding p-values are reported in the tables. A p-value of <0.05 was considered statistically significant.

## Results

Among the 240 participants, both VFA and SFA were significantly higher in individuals with T2DM compared to those without diabetes. No statistically significant differences were noted between males and females within the diabetic or non-diabetic groups for either VFA or SFA. However, across all age groups, patients with diabetes consistently demonstrated higher fat accumulation. Age-stratified analysis also revealed significant differences in VFA and SFA between the two groups, with fat volumes generally increasing with age, particularly in the diabetic cohort. The mean age was recorded to be 46.47 and 42.60 years in the diabetic and non-diabetic subjects, respectively, with a slight male predominance (53.33%) amongst diabetic patients, whereas there was female (51.67%) predominance amongst the patients without diabetes, as shown in Table 1.

**Table 1:** Gender distribution among individuals with and without diabetes DM: Diabetes mellitus; Data are presented as n (%).

Gender	DM (n=120)	Non-DM (n=120)
Male	64 (53.33%)	58 (48.33%)
Female	56 (46.67%)	62 (51.67%)
Total	120 (100.0%)	120 (100.0%)

Although a slight variation in gender distribution was noted between groups, the difference was minimal. To address potential gender-related differences, subgroup analyses for VFA and SFA were presented separately for males and females. The mean HbA1c was recorded as 6.57 and 5.38%, and the mean BMI was recorded to be 25.63 and 23.62 kg/m^2^ in the diabetic and non-diabetic groups, respectively, as illustrated in Table 2.

**Table 2:** HbA1c, BMI, VFA, and SFA levels in individuals with and without diabetes mellitus (DM) HbA1c: Glycated hemoglobin; BMI: Body mass index; VFA: Visceral fat accumulation; SFA: Subcutaneous fat accumulation; SD: Standard deviation

Parameters (mean±SD)	DM	Non-DM
HbA1c (%)	6.57±0.605	5.38±0.13
BMI (kg/m^2^)	25.63±1.51	23.62±1.47
VFA (cm^3^)	3075.72±835.60	2042.54±641.00
SFA (cm^3^)	3303.39±948.26	1795.75±561.14

The mean VFA levels were recorded as 3075.72 cm^3^ and 2042.54 cm^3^ in patients with and without diabetes, respectively, and the difference was statistically significant (p<0.00001), as determined by the unpaired Student's t-test. This difference remained statistically significant even after adjusting for BMI, age, and sex. Furthermore, the mean VFA was recorded as 3028.33 cm^3^ and 2028.13 cm^3^ in male patients with and without diabetes, whereas the mean VFA was 3119.86 cm^3^ and 2056.03 cm^3^ in women with and without diabetes, respectively, as illustrated in Table 3.

**Table 3:** Genderwise distribution of VFA in individuals with and without diabetes mellitus (DM) VFA: Visceral fat accumulation; SD: Standard deviation

Gender	VFA in DM (cm^3^)	VFA in Non-DM (cm^3^)	U-value	P-value
Males (mean±SD)	3028.83±774.52	2028.13 ± 624.07	2312.5	<0.00001
Females (mean±SD)	3119.86±886.05	2056.03 ± 661.25	2450.0	<0.00001
P-value (M vs F)	-	-	U=3880.0 (DM)	0.2781
			U=3955.0 (Non-DM)	0.4000

**Figure 1:**
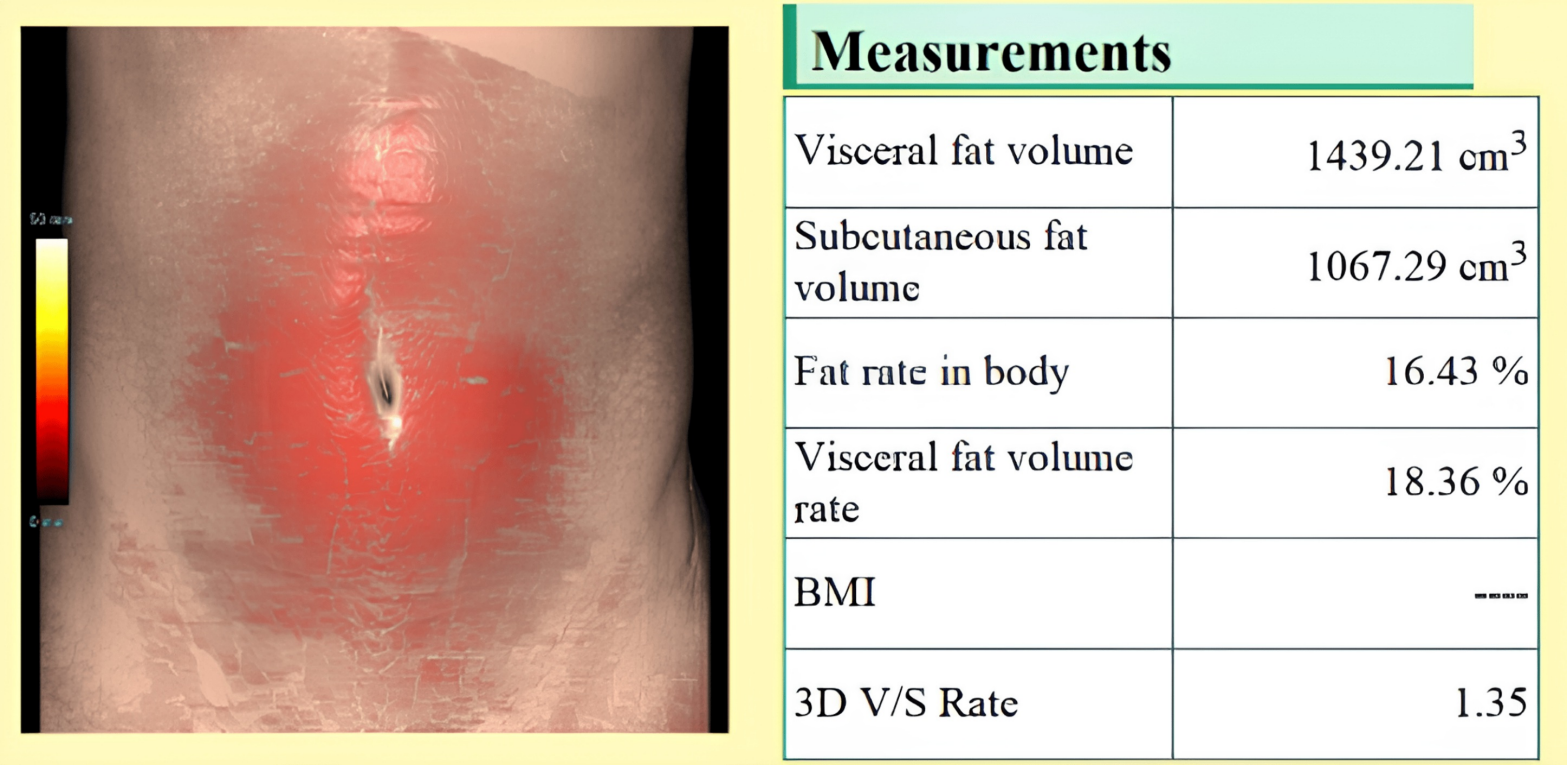
Report provided by the fat analyzer application from the Synapse software depicting VFA and SFA in an individual with diabetes VFA: Visceral fat accumulation; SFA: Subcutaneous fat accumulation

**Figure 2:**
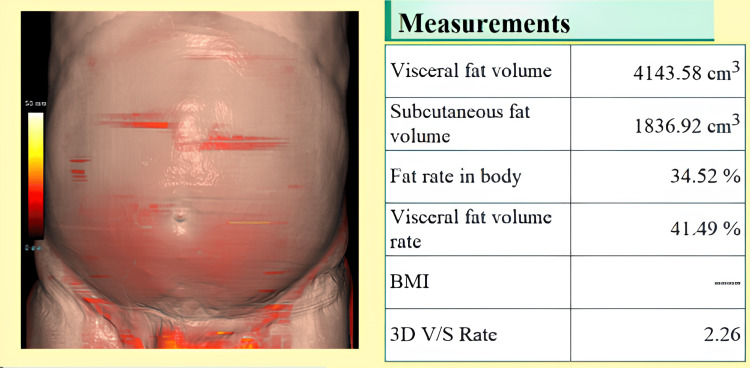
Report provided by the fat analyzer application from the Synapse software depicting VFA and SFA in an individual with diabetes VFA: Visceral fat accumulation; SFA: Subcutaneous fat accumulation

However, there was no statistically significant difference between the male and female patients in the diabetic and non-diabetic groups. This finding suggests that the relationship between diabetes and visceral fat is not sex-dependent in this population, which may inform gender-neutral screening thresholds for visceral adiposity-related metabolic risk. The report generated by the fat analyzer for one individual from the diabetic and non-diabetic groups is depicted in Figures 1, 2.

Upon assessing the mean VFA, statistically significant difference between the diabetic and non-diabetic groups was observed across all age groups, as illustrated in Table 4.

**Table 4:** Distribution of VFA across age groups in individuals with and without diabetes mellitus (DM) VFA: Visceral fat accumulation; SD: Standard deviation. VFA values in mean±SD

Age group (years)	VFA in DM (cm^3^)	VFA in Non-DM (cm^3^)	U-value	P-value
18–30	3009.87±1113.42	1983.01±591.73	420.0	0.00062
31–45	2997.53±856.86	2136.44±652.68	390.0	<0.00001
46–60	3311.63±654.71	2013.46±720.50	310.5	<0.00001
>60	2574.17±947.62	1810.24±543.25	485.0	0.0244
P-value (Kruskal-Wallis)	-	-	H=6.55 (DM)	0.089
			H=0.78 (Non-DM)	0.463

We found no statistically significant differences between patients who were diabetic and those who were non-diabetic. Notably, patients in the diabetic group aged >60 years exhibited relatively lower VFA levels compared to younger patients with diabetes, a finding that may reflect age-related changes in fat distribution or survival bias.

The mean SFA levels were recorded as 3309.39 cm^3^ and 1795.75 cm^3^ in patients with and without diabetes, which was found to be statistically significant. The mean SFA levels in male participants with and without diabetes were 3232.53 cm^3^ and 1795.95 cm^3^, whereas they were 3370.62 cm^3^ and 1795.53 cm^3^ in female participants, respectively. This result was found to be statistically significant, as illustrated in Table 5.

**Table 5:** Genderwise distribution of SFA in individuals with and without diabetes mellitus (DM) SFA: Subcutaneous fat accumulation; SD: Standard deviation. Values are in mean±SD

Gender	SFA in DM (cm^3^)	SFA in Non-DM (cm^3^)	U-value	P-value
Males	3232.53±960.00	1795.98±541.02	2190.0	<0.00001
Females	3370.62±911.57	1795.53±583.75	2230.0	<0.00001
P-value (M vs F)	-	-	U=3765.0 (DM)	0.2138
			U=3920.0 (Non-DM)	0.4270

However, there was no statistically significant difference between the male and female patients in the diabetic and non-diabetic groups. While subcutaneous fat is traditionally considered metabolically less harmful than visceral fat, the substantial difference in SFA observed between individuals with and without diabetes in this study suggests a potentially more complex role in this population. Notably, we observed no statistically significant difference in SFA between male and female participants within both diabetic and non-diabetic groups, indicating that the relationship between diabetes and subcutaneous fat is largely sex-independent in this cohort. This contrasts with findings from Western populations, where female subjects typically accumulate more subcutaneous fat than male subjects. Such divergence may reflect ethnic or regional differences in fat distribution patterns and highlights the need for further research into the metabolic implications of SFA, particularly in the South Asian populations.

On assessment of the mean SFA across both groups, statistically significant difference between individuals with and without diabetes was observed across all age groups, as illustrated in Table 6.

**Table 6:** Distribution of SFA across age groups in individuals with and without diabetes mellitus (DM) SFA: Subcutaneous fat accumulation; SD: Standard deviation. Values are in mean±SD.

Age group (years)	SFA in DM (cm^3^)	SFA in Non-DM (cm^3^)	U-value	P-value
18–30	3942.76±823.16	1780.89±593.28	380.0	<0.00001
31–45	3227.35±964.87	1841.35±541.76	345.0	<0.00001
46–60	3471.44±851.02	1787.08±636.95	310.0	<0.00001
>60	2446.40±797.27	1646.87±491.36	455.0	0.0094
P-value (Kruskal-Wallis)	-	-	H=8.99 (DM)	0.0030
			H=0.45 (Non-DM)	0.8100

Statistically significant difference was observed across all age groups among individuals with diabetes; however, no significant differences were found among those without diabetes.

## Discussion

This study demonstrates that both VFA and SFA, measured by CT imaging, are significantly higher in individuals with T2DM compared to individuals without diabetes in a rural population from South India. The association held true across most age groups and both sexes. Notably, SFA, which is often considered metabolically benign, showed a strong correlation with diabetes, suggesting its potential utility as an adjunct marker. These findings reinforce the importance of regional fat distribution, particularly VFA and SFA, as early predictors of diabetes, even in resource-limited rural settings. Metabolic disorders are difficult to manage if not diagnosed early. Metabolic conditions frequently contribute to or coexist with other metabolic diseases, indicating a shared pathophysiological basis. The age of the patient is an important parameter, as it reflects physical activity and lifestyle in terms of fat accumulation. Over the years, there has been a paradigm shift wherein younger people suffer from cardiovascular diseases, compared to the scenario observed in previous decades. This has largely been due to a sedentary lifestyle, with increased obesity posing a higher risk for cardiovascular diseases.

Our retrospective study involved 240 individuals with a mean age of 46.47 and 42.60 years among patients with and without diabetes, respectively. These are notably younger compared to comparable studies conducted in other populations. This may reflect unique lifestyle and environmental factors prevalent in rural South India, including earlier onset of T2DM due to genetic predisposition, dietary patterns high in carbohydrates and fats, and reduced physical activity related to socioeconomic changes. Several studies have reported that South Asians tend to develop diabetes at a younger age and lower BMI compared to Western populations, emphasizing the need for earlier screening and preventive strategies in this demographic. This younger mean age underscores the clinical importance of identifying reliable markers like VFA and SFA for timely diagnosis and management. Zhou et al. [10] also recorded a mean age of 45.47 and 59.21 years among individuals with and without diabetes, respectively. Yokokawa et al. [3], in their study, reported that the mean age of patients without diabetes and with diabetes was 60.1 and 66.1 years, respectively. Other researchers, such as Luo et al. [1] and Fukuda et al. [8] reported the respective mean age to be 55.9 and 64 years. In our study, we found a slight male predominance (53.33%) among patients with diabetes, whereas there was a female predominance (51.67%) among those without diabetes.

Our study results are in agreement with those of Zhou et al. [10], who also reported a male predominance among patients with diabetes and a female predominance among patients without diabetes. Yokokawa et al. [3] reported about 52% of male patients without diabetes and 80% of male patients with diabetes in their study, which was also observed by Luo et al. [1]. However, contrasting observations were made by Fukuda et al. [8] and Tripathi et al. [9], with 45% and 46.3% of male patients with diabetes, respectively.

The mean HbA1c and BMI values in the diabetic group were 6.57% and 25.63 kg/m^2^, whereas they were 5.38% and 23.62 kg/m^2^ in the non-diabetic group, respectively; and this result was found to be statistically significant. The observed difference in BMI between the diabetic and non-diabetic groups may act as a confounding factor in assessing the relationship between fat distribution and diabetes. Higher BMI is itself a known risk factor for altered fat distribution and insulin resistance. While our study did not perform multivariate adjustment to isolate the independent effects of VFA and SFA apart from BMI, future studies with more comprehensive statistical modeling are warranted to clarify these relationships. Controlling for BMI would help determine whether fat distribution predicts diabetes risk beyond the influence of general adiposity. Similar observations were reported by Zhou H et al. [10], who found HbA1c and BMI in patients with diabetes to be 6.91% and 32.67 kg/m^2^, whereas in patients without diabetes, it was 5.38% and 28.28 kg/m^2^. Yokokawa et al. [3] and Shrestha et al. [11] reported that patients with diabetes had a significantly higher mean BMI, waist circumference, and VFA than those without diabetes, which is also in agreement with Fukuda et al [8]. However, Tripathi et al. [9] reported significant differences in such levels, wherein HbA1c and BMI values in patients with diabetes were 8.1% and 31.7 kg/m^2^ and were 5.5% and 27.7 kg/m^2^ in individuals without diabetes, respectively.

Fat distribution is affected by sex hormone levels, ageing, and genetic factors [12]. Visceral fat build-up is considered a risk factor for cardiovascular illnesses, including diabetes. This association is particularly significant as visceral adiposity confers an elevated cardiovascular risk even among patients with established major adverse cardiovascular events. MetS is a metabolic condition that indicates who is more prone to developing cardiovascular diseases due to insulin resistance [3,13]. Visceral fat build-up, a key hallmark of MetS, is intimately linked to insulin resistance and recognized as a distinct risk indicator for T2DM. Visceral fat area is defined as a waist circumference of ≥85 cm^2^ in men and ≥90 cm^2^ in women, resulting in a visceral fat area of 100 cm^2^ in an abdominal CT scan at the umbilical level [3,13].

The mean VFA was 3075.72 cm^3^ and 2042.54 cm^3^ in patients with and without diabetes, and this difference was statistically significant. While significant differences in visceral fat volume were observed between diabetic and non-diabetic groups overall, no significant intragroup differences were found when comparing subgroups stratified by age or sex within each group. This suggests that age and sex did not significantly influence visceral fat volume within the diabetic or non-diabetic cohorts. It should be noted that these values represent visceral fat volume (cm³) obtained from volumetric CT analysis rather than the commonly reported visceral fat area (cm²) measured at a single axial slice at the umbilical level. Our volumetric approach involves summing cross-sectional areas across multiple slices to provide a more comprehensive estimation of total visceral fat. While standard clinical cutoffs typically use cm² values, volumetric measurements may better reflect total fat burden. For reference, future studies could explore standardizing volume-to-area conversions to facilitate comparison. On further assessment, we found the mean VFA levels to be 3028.33 cm^3^ and 2028.13 cm^3^ in male patients with and without diabetes, respectively, whereas it was 3119.86 cm^3^ and 2056.03 cm^3^ in female participants. There were no statistically significant differences between male and female patients in the diabetic and non-diabetic groups. When we compared the VFA with age, we found significant differences between patients with and without diabetes in all age groups. However, no significant difference was observed between the diabetic and non-diabetic groups. A similar study by Tripathi et al. [9], who evaluated the visceral fat in patients with diabetes and those without, reported that visceral fat scores in patients with diabetes were almost double those observed in individuals without diabetes. Additionally, Yokokawa et al. [3], Luo et al. [1], and Shrestha et al. [11] reported a higher visceral fat area in individuals with diabetes than those without. Kim et al. identified the optimal visceral fat area cut-off values for diagnosing T2DM in the Korean population as 118.8 cm² for men and 82.6 cm² for women [14]. In a study of Japanese Americans, Wander et al. [15] found that the baseline cut-off value for intra-abdominal fat area (IFA) was 102.7 cm² in those who developed diabetes, and 74.3 cm² in others who did not. After adjusting for confounding factors, a one standard deviation increase in IFA was associated with a 1.65-fold higher risk of developing diabetes over 10 years [14,15].

Body fat distribution in humans differs according to gender [12]. For instance, oestrogen promotes fat accumulation in the gluteofemoral subcutaneous adipose tissue (SAT) rather than in the visceral adipose tissue (VAT). A study on fatty acid kinetics using radiolabelled triolein showed that premenopausal women stored a greater proportion of dietary fat in the SAT than men [12,16]. After menopause, women tend to store more fat centrally than they did before menopause [12,17,18]. Additionally, oestrogen replacement therapy in postmenopausal women has been shown to reduce VAT and BMI [12,19]. Studies have shown that VFA has considerably more negative consequences than SFA and remains an independent risk factor for diabetes [9,20]. A recent study found that visceral fat is an unbiased marker of diabetes mellitus [3,9].

Even researchers such as Wu et al. [21], Liu et al. [22], Chen et al. [23], and Neel et al. [24] agree that visceral adiposity remains associated with diabetes. In our study, the mean SFA level was 3309.39 cm^3^ and 1795.75 cm^3^ in diabetic and non-diabetic individuals, respectively, and this difference was statistically significant. While visceral fat is generally considered a stronger predictor of diabetes, recent studies suggest that subcutaneous fat also contributes to metabolic risk, possibly through inflammatory processes. However, it is important to consider whether differences in subcutaneous fat are independent of overall body fat or BMI, which was not controlled for in this study. On further assessment, we found the mean SFA level in male patients who were diabetic and non-diabetic to be 3232.53 cm^3^ and 1795.95 cm^3^, whereas it was 3370.62 cm^3^ and 1795.53 cm^3^ in women, respectively; and this was found to be statistically significant. However, there was no statistically significant difference (intragroup comparison) between male and female individuals in the diabetic and non-diabetic groups. When correlating the age of the patients with SFA, we found a statistically significant difference between individuals with and without diabetes across all age groups. We also found a statistical significance across all diabetic age groups (intragroup comparison); however, it was not significant in non-diabetic individuals. This finding contrasts with traditional fat distribution patterns where sex differences are commonly observed, possibly indicating unique fat deposition characteristics in the rural Indian population studied. Such distinctions may be important when tailoring clinical risk thresholds for metabolic diseases in this demographic. Our findings align with those of Shrestha et al. [11] who observed elevated stem cell factor levels in individuals with diabetes compared to those without diabetes. Similarly, Yokokawa et al. [3] reported that the mean SFA was significantly higher in women with diabetes than in their non-diabetic counterparts, while no significant difference was noted among men [8,11]. We observed significant differences in VFA and SFA between patients with and without diabetes; however, these differences were not observed across various ages and sexes, suggesting that both VFA and SFA are reliable indicators of diabetes mellitus. This intriguing pattern may reflect age-related changes in subcutaneous fat function among patients with diabetes, possibly due to chronic low-grade inflammation or diminished fat-buffering capacity that alters fat metabolism. In contrast, individuals with diabetes may maintain more stable SFA distribution with age. However, we recommend the use of SFA as an adjunct to VFA until further substantial supporting evidence is added, which may project and prove SFA as a reliable standalone marker.

In addition, although we assessed all the parameters according to the requirements of our study, we encountered some limitations, including a limited sample size and a single-center, non-randomized study design without multiple observers for procedure evaluation. Furthermore, residual confounding may still be present due to the lack of adjustment for important variables such as dietary intake, physical activity levels, and socioeconomic status, all of which are known to influence fat distribution and metabolic risk. Future studies should incorporate these factors to better isolate the effects of visceral and subcutaneous fat on diabetes risk. Therefore, for future studies, we recommend a larger sample size, and a multicenter, randomized, multi-observer, standardized study to overcome the shortcomings of our study.

## Conclusions

CT-based measurements of VFA and SFA provide meaningful predictive insights into the risk of T2DM in a rural Indian population. Our study demonstrated significant differences in both VFA and SFA between patients with and without diabetes, highlighting the important alterations in fat distribution associated with the disease. While VFA is well-established as a reliable marker for metabolic and cardiovascular risk, evidence supporting the independent predictive value of SFA remains limited. Therefore, we recommend using SFA as an adjunct to VFA in clinical risk assessment until further large-scale studies validate its role as a standalone marker. These findings underscore the importance of regional fat distribution assessment in diabetes risk stratification and suggest that incorporating CT-based fat quantification could improve early diagnosis and personalized management strategies in rural Indian populations. Future research with larger, multicentric cohorts is warranted to confirm these findings and to explore potential thresholds tailored for this demographic.
